# Cerebellar engagement in the attachment behavioral system

**DOI:** 10.1038/s41598-022-17722-x

**Published:** 2022-08-09

**Authors:** Eleonora Picerni, D. Laricchiuta, F. Piras, L. Petrosini, G. Spalletta, D. Cutuli

**Affiliations:** 1 IRCCS Santa Lucia Foundation, Via Ardeatina 306, 00179 Rome, Italy; 2 Sapienza University, Via dei Marsi 78, 00185 Rome, Italy; 3 Division of Neuropsychiatry, Menninger Department of Psychiatry and Behavioral Sciences, Baylor College of Medicine, Houston, TX 77030 USA

**Keywords:** Neuroscience, Cognitive neuroscience

## Abstract

Brain structural bases of individual differences in attachment are not yet fully clarified. Given the evidence of relevant cerebellar contribution to cognitive, affective, and social functions, the present research was aimed at investigating potential associations between attachment dimensions (through the Attachment Style Questionnaire, ASQ) and cerebellar macro- and micro-structural measures (Volumetric and Diffusion Tensor Imaging data). In a sample of 79 healthy subjects, cerebellar and neocortical volumetric data were correlated with ASQ scores at the voxel level within specific Regions Of Interest. Also, correlations between ASQ scores and age, years of education, anxiety and depression levels were performed to control for the effects of sociodemographic and psychological variables on neuroimaging results. Positive associations between scores of the Preoccupation with Relationships (ASQ subscale associated to insecure/anxious attachment) and cortical volume were found in the cerebellum (right lobule VI and left Crus 2) and neocortex (right medial OrbitoFrontal Cortex, OFC) regions. Cerebellar contribution to the attachment behavioral system reflects the more general cerebellar engagement in the regulation of emotional and social behaviors. Cerebellar properties of timing, prediction, and learning well integrate with OFC processing, supporting the regulation of attachment experiences. Cerebellar areas might be rightfully included in the attachment behavioral system.

## Introduction

Being the human nature inherently rooted in its social interactions, intersubjective relationships play a crucial role in survival and reproduction processes and support the development and preservation of physical and mental health^1^. As infants, humans strongly rely on others and have fundamental psychological needs for safety and acceptance^2^. The attachment theory^2–4^ posits that humans are endowed with an innate behavioral attachment system which may elicit the attention of, and support from, other significant persons, the attachment figures. Thus, the attachment to others is considered a motivational system associated with resistance to separation, and grief and disruption when loss of a close relationship occurs^3^. The attachment drive is triggered by psychological or physiological threats and leads to seek proximity to the attachment figure to get protection and restore emotional balance^5^.

According to attachment theory, from early social interactions with significant primary figures the child develops distinct mental representations of the self and others that become part of general interpersonal schemata of the individual (“*internal working models”*^5^), support social development, and influence thoughts, feelings, and behaviors throughout the lifespan. Namely, infant attachment orientations are retained to influence adults’ social, emotional, and affective relationships^3,6^ with romantic partners or close friends, or even unfamiliar persons^7^. Therefore, the individual’s attachment history is associated with individual differences in emotional and cognitive mechanisms^8^*.* Traditionally, attachment has been categorized into three main styles—secure, anxious, and avoidant attachment^3^, to which a fourth one—disorganized attachment—has been then added^9^.

Children with *secure* attachment are confident of caregivers’ support and use adult attachment figures as a secure base for exploration; when adults, securely attached individuals may enjoy intimate relationships, seek out social support, share feelings with other people, and be not worried about abandonment. Children with *anxious/ambivalent* attachment are exaggeratedly distressed by separation; when adults, anxiously attached individuals may exhibit high levels of worry and impulsiveness in their relationships and have generalized feelings of abandonment and rejection. Children with *avoidant* attachment seem undisturbed by the separation from the caregivers; as adults, avoidantly attached individuals may view themselves as self-sufficient, seek less intimacy with partners, do not care about close relationships, and tend to suppress their emotions^2,3^. Finally, children and adults with *disorganized* attachment do not express consistent attachment behavior, lack a coherent approach towards relationships, and may show mixed responses of anxiety and avoidance. In conclusion, the attachment theory provides a theoretical framework for the development of fundamental individual schemata that in adults may influence the quality and quantity of close interpersonal relationships.

Despite its heuristic value, attachment theory has been criticized for the failure to incorporate temperamental factors as well as social and cultural variability^10^. Empirical data on stability of individual attachment across the lifespan are conflicting and correlations between infancy and adulthood attachment measures are reported to be small to moderate^11,12^. Notably, major transforming life events occurring after infancy, such as childbirth, could affect one's attachment strategies^13–15^. As for intergenerational transmission of attachment relationships, evidence for continuity of attachment from mother to infant is more robust for secure attachment than for insecure attachment^16^.

Moreover, despite attachment theory has generated extensive research in social and clinical psychology (see reviews by^5^^,^^17^), the brain structural bases of the individual differences in attachment style are not yet fully clarified.

To date, a number of studies indicated the association of attachment-related emotional states with neurobiological and genetic substrates^8,18–20^. Furthermore, attachment styles appear to covary with brain morphometry measures, as cortical thickness and gray matter volume of specific cerebral regions (orbito-frontal cortex, anterior temporal pole, hippocampus, fusiform gyrus, cingulate cortex, insula, amygdala, striatum)^21–24^. Consistently, functional imaging studies reported attachment-modulated activations in fronto-striatal-limbic circuits during social and affective processing and regulation tasks (involving emotional and cognitive mentalization)^25^. However, few studies have investigated whether differences in attachment styles are associated even with cerebellar gray matter modifications^26–28^, despite the growing evidence of the relevant cerebellar contribution (modulatory rather than generative) to cognitive, affective, and social functions^29^.

On this basis, the present research was aimed at investigating the potential associations between attachment styles (assessed by the Attachment Style Questionnaire, ASQ^30^) and cerebellar macro- and micro-structural measures. In a clinically healthy sample of 79 subjects of both sexes, at macro-structural level the volumetric variations were analyzed through Region Of Interest (ROI)-based analyses, and at micro-structural level, through a Diffusion Tensor Imaging (DTI) protocol.

We found a significant positive association between an ASQ subscale (Preoccupation with Relationships*)* and volumes of the cerebellar right Lobule VI and left Crus 2. We next extended our analyses to the associations between attachment styles and volumes of the main cortical sites which the attachment-associated cerebellar areas projected to. In fact, the cortico-cerebello-cortical system comprises a series of closed modular ‘loops’, each of which shares a specific isomorphic organization in which cortical areas project to specific areas of the cerebellar cortex via the pontine nuclei, and in turn receive projections from these areas via cerebellar dentate nucleus and thalamus^31–33^. Anticipating the results, we found that the right medial Orbito-Frontal Cortex (mOFC, BA11), a prefrontal region receiving projections from Crus 2, was significantly associated with the ASQ subscale previously quoted.

## Results

### Sociodemographic and psychological variables

Mean scores and standard deviations of each psychological variable: ASQ subscales, Hamilton anxiety rating scale (indicated in the text as HAM-A) and Hamilton depression rating scale ‐17 items (HAM‐D17, indicated in the text as HAM-D) are reported in Table 1.

**Table 1:** Scores on psychological instruments (ASQ subscales, HAM-A and HAM-D) for all subjects, males and females (mean ± standard deviation).

ASQ subscale	All participants	Males	Females
Confidence	34.35 ± 4.73	34.03 ± 4.31	34.63 ± 5.09
Discomfort with Closeness	34.15 ± 6.65	33.42 ± 6.20	34.77 ± 7.01
Relationships as Secondary	14.09 ± 5.18	15.47 ± 4.18	14.09 ± 5.18
Need for Approval	18.73 ± 5.75	18.06 ± 5.66	19.30 ± 5.83
Preoccupation with Relationships	25.24 ± 6.42	25.5 ± 7.36	25.02 ± 5.59
HAM-A	4.96 ± 3.86	4.16 ± 3.79	5.26 ± 3.82
HAM-D	3.00 ± 2.81	2.41 ± 2.83	3.48 ± 2.74

While age, gender, and education years were not significantly related to any ASQ subscale, HAM-A and HAM-D showed a number of significant associations with ASQ subscales. Namely, significant positive associations between the scores on Need for Approval subscale and HAM-A and HAM-D data, as well as between scores of Relationships as Secondary subscale and HAM-A data were found (Table 2).

**Table 2:** ASQ subscales and sociodemographic variables.

ASQ subscales	Age		Years of education		Gender		HAM-A		HAM-D	
	*r*	*p*	*r*	*p*	*t*	*p*	*r*	*p*	*r*	*p*
Confidence	0.01	0.90	− 0.02	0.87	− 0.56	0.58	− 0.15	0.18	− 0.22	0.05
Discomfort with Closeness	0.09	0.43	− 0.07	0.51	− 0.90	0.37	0.02	0.81	0.13	0.22
Relationships as Secondary	0.17	0.14	− 0.06	0.59	1.28	0.20	**0.25**	**0.02**	0.21	0.06
Need for Approval	0.02	0.87	0.11	0.33	− 0.96	0.34	**0.39**	** < 0.0001**	**0.31**	** < 0.01**
Preoccupation with Relationships	0.01	0.90	0.09	0.43	− 0.33	0.74	0.21	0.06	0.11	0.30

Significant results (*p* < 0.05) are in Bold.

As expected, significant direct correlations were found between ASQ subscales, except for the not significant correlation between Discomfort with Closeness and Preoccupation with Relationships (Table 3).

**Table 3:** Correlations between ASQ subscales.

	C		DwC		RaS		NfA		PwR	
C			− 0.54	*p* < 0.0001	− 0.53	*p* < 0.0001	− 0.47	*p* < 0.0001	− 0.29	*p* = 0.01
DwC	− 0.54	*p* < 0.0001			0.31	*p* = 0.006	0.33	*p* = 0.003	0.20	*p* = 0.073
RaS	− 0.53	*p* < 0.0001	0.31	*p* = 0.006			0.50	*p* < 0.0001	0.33	*p* = 0.003
NfA	− 0.47	*p* < 0.0001	0.33	*p* = 0.003	0.50	*p* < 0.0001			0.66	*p* < 0.0001
PwR	− 0.29	*p* = 0.01	0.20	*p* = 0.073	0.33	*p* = 0.003	0.66	*p* < 0.0001		

Abbreviations: C: Confidence; DwC: Discomfort with Closeness; RaS: Relationships as Secondary; NfA: Need for Approval; PwR: Preoccupation with Relationships.

Significance for results *p* < 0.05. Correlation between PwR and DwC was not significant.

### ROI-based VBM

**Figure 1:**
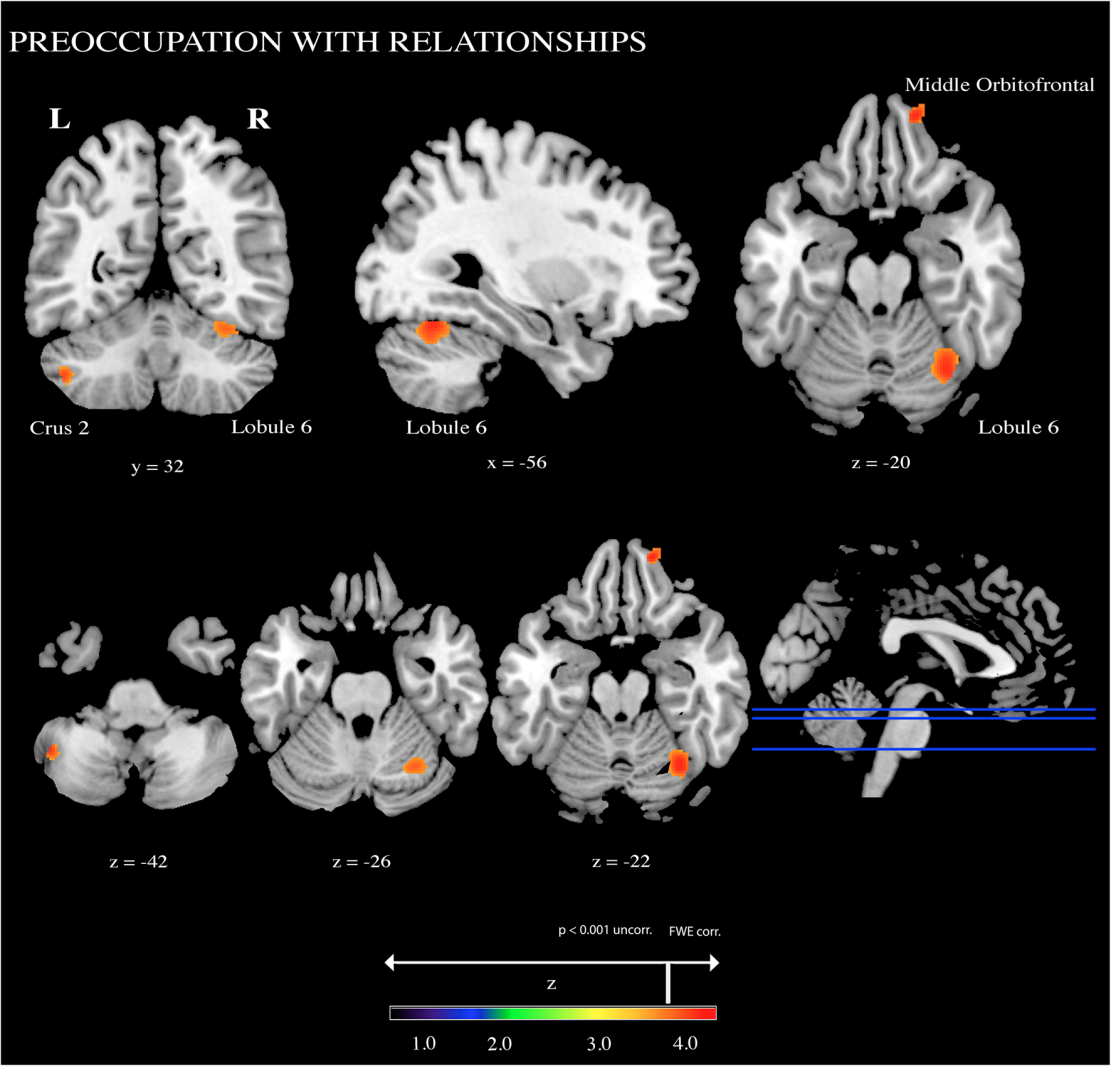
Positive associations between a priori Regions Of Interest (ROIs) and Preoccupation with Relationships ASQ subscale. Coordinates are in Montreal Neurological Institute (MNI) space. Z above colorbar indicates normalized t-values. In figure left is left.

Analyses on the cerebellar areas revealed significant positive associations between the Preoccupation with Relationships ASQ subscale and extended clusters in right lobule VI (390 voxels) (p_FWEcorr_ = 0.044) and in left Crus 2 (84 voxels) (p_FWEcorr_ = 0.037) (Fig. 1; Table 4).

**Table 4:** Regional gray matter volumes (ROI-based analyses) and Preoccupation with Relationships ASQ subscale.

Label for peak direction	Side	Extent (n voxels)	t	*p*	equivZ	x,y,z (mm)	Effect size (d)
**Preoccupation with relationships**							
Cerebellum Crus 2 ↑	L	84	3.97	**0.037**	3.77	− 45, − 52, − 45	0.73
Cerebellum Lobule VI ↑	R	390	3.91	**0.044**	3.72	32, − 61, − 20	0.70
Middle Orbitofrontal Cortex ↑	R	113	3.78	**0.048**	3.60	16, 54, − 24	0.45

Abbreviations: *p* = significance at the peak level. L = left. R = right.

Coordinates are in Montreal Neurological Institute (MNI) space.

Significant values (FWE corrected) are in bold.

Furthermore, the Preoccupation with Relationships ASQ subscale was positively correlated with a cluster (113 voxels) in right mOFC (BA11) (p_FWEcorr_ = 0.048) (Fig. 1; Table 4). Then, mean values from significant clusters were extracted and then correlated with the Preoccupation with Relationships subscale scores, in order to calculate effect sizes of the results. Effect sizes values (basing on Cohen^34^ and Hattie^35^) are reported in Table 4.

### DTI analyses

MD and FA values of cerebellar and cortical areas failed to reveal any significant association with Preoccupation with Relationships ASQ subscale.

## Discussion

Neuroimaging studies on the associations between neurobiological measures in specific brain regions and attachment styles have only occasionally reported the involvement of cerebellar circuits^26–28,36–38^, and often have described it almost as an anecdotical finding. The present research, specifically aimed at analyzing the participation of cerebellar regions in the attachment behavioral system, found significant positive associations between the scores of the Preoccupation with Relationships, an ASQ subscale associated to insecure/anxious attachment, with extended clusters in cerebellar (right lobule VI and left Crus 2) and cortical (right mOFC) regions. The increased cerebellar and cortical volumes were not accompanied by modifications in DTI values. Although some studies^39,40^ emphasize the relationship between anxiety related personality characteristic in healthy subjects and DTI-derived indices of WM and GM integrity, most of studies have described the relationship between anxiety related characteristics and micro-structural abnormalities in individuals with various neuropsychiatric disorders, such as obsessive compulsive disorder, Tourette’s syndrome and autism^41–43^, as well as in attachment-related pathological conditions^44,45^.

Noteworthy, in front of a very limited number of reports describing DTI variations in relation to personality/contextual factors, the present research is the first report trying to address GM micro-structural data in relation to attachment individual differences in non-pathological conditions.

Surely working with larger sample might help detecting more subtle individual differences, to date it is possible to advance that only macro-structural integrity of certain structures contributes to explain the biological variance which leads to personality phenotypes, such as the attachment system.

The positive associations between Preoccupation with Relationships scores and the highlighted brain volumes are consistent with both the emotional and social processing, because of the increased efforts at processing the emotional stimulus and the hypervigilant nature of individuals with anxious/preoccupied attachment. In fact, each attachment style is characterized by a specific emotional and cognitive pattern. In particular, anxious/preoccupied people are characterized by intense emotional responses and sustained search for security/predictability in the relationships. Despite their strong desire to achieve intimacy and approval in relationships, their cognitive order is characterized of low opinion of themselves as deserving of salient relationship, and they are mistrustful of others and their availability, and anxiously expect rejection or abandonment by relationship partners^5,46^.

Considering the property of cerebellar networks in building internal models of internal or external environments through signal error processing^47^, anxiously attached subjects could display continuous error signals to the cerebellum that thus does not habituate^48^. These unremitting inputs could provoke a compensatory increase, leading to an enlargement in cerebellar volumes.

Although healthy, all participants were also evaluated by HAM-D and HAM-A scales^49,50^. Positive correlations were found between scores of Need for Approval ASQ subscale and both HAM scales, and between scores of Relationships as Secondary ASQ subscale and HAM-A scale. Thus, the more anxious and depressive tendencies were evident, the more insecure attachment patterns were present, once more indicating that emotional reactions are modulated by individual differences in the social bonding^5^.

Our present findings fit with previous structural data describing that anxious attachment is associated with increased volumes in cerebellar areas^26,28^ and lateral orbital gyrus^26,51^. Also, they fit with functional data describing that anxiously attached adults display enhanced activation to positive approach-related facial expression in the cerebellar and prefrontal areas involved in perception of facial emotion, assessment of affective value and social distance^27,28^. Interestingly, enhanced cerebellar activation was observed in adolescents with a high negativity of the self-model, typical for the anxious attachment dimension^37^. Furthermore, increased cerebellar activation has been described in a study investigating grief through the exposure of bereaved women to pictures of their deceased loved one^52^. Bowlby^4^ viewed grief related to affective loss as a natural expression of the attachment behavioral system evoked to discourage prolonged separation from a primary attachment figure. Such a kind of grief implies the coordination of multiple functions, as affect processing, mentalizing, episodic memory retrieval, processing of familiar faces, visual and motor imagery, autonomic regulation, automatic motor responses. Notably, most of these functions are mediated by a distributed neuronal network of which the cerebellum (especially, its posterior regions) is part^53^. Cerebellar areas then might be rightfully inserted in the attachment behavioral system described by Bowlby^2^. The cerebellar contribution to the attachment system may be interpreted as concomitant to a “feeling of being drawn toward” the affective stimulus, and reflects the more general cerebellar engagement in regulation of emotional and social behaviors^29,55–57^. Given the neuronal circuits putatively responsible for social processes are closely associated with, and virtually inextricable from, those devoted to emotional regulation^58^, it is not surprising that the same regions of the posterior cerebellum and prefrontal cortex are involved in both emotional regulation and social interaction.

According to psychological models of adult attachment^3^, the complex interactions of thoughts and behaviors required for sensitive parenting of offspring enable formation of individual’s first social bonds, critically shape infants’ behavior, and deeply influence the adult social behavior. Such an assumption is strongly supported by animal and human studies indicating that early attachment experiences influence brain development and may result in permanent structural and functional brain changes and in individual differences in cognitive performance and social behavior^19,59–61^. In fact, in rodents, maternal experiences exert a marked transgenerational impact and influence offspring’s phenotype at behavioral (learning and memory abilities, attentional performance, coping response to stress, social behavior, anxiety levels) and neurobiological (synaptic plasticity, methylation in frontal and hippocampal areas, hippocampal neurogenesis, striatal and cerebellar neurotrophins) levels^62,63^. Noteworthy, the first mother-infant relationships influence not only infant’s developmental processes, but also mother’s neurobiological and behavioral processes. A recent study^64^ on the maternal brain functional connectivity in the early postpartum phases reports changes in cerebello-cortical connectivity associated with changes in maternal anxiety toward her child, providing insight into the mother-infant bond in the specific context of anxiety. Analogously, fMRI studies on maternal brain during processing of infant affective cues have repeatedly implicated the cerebellum^65–67^, suggesting that enhanced cerebello-cortical connectivity may increase prioritization of processing infant cues in the maternal brain. Very recently, in child-rearing mothers it has been described a significant association of increased resting-state functional activity in lobule VI with increased maternal trait anxiety and poorly adaptive sensory processing^68^. Such a finding has been interpreted as an indicator of maternal trait anxiety and risk of parenting stress. The positive association between volumes of lobule VI-Crus 2 and Preoccupation with Relationships scores reported in the present research is consistent with neuroimaging findings^69^ describing the activation of cerebellar and neocortical areas belonging to the default mode network that regulates the switch from an internal reference state to external target-oriented behaviors, once more emphasizing the cerebellar role of interface between internal and external environments. Since lobule VI-Crus 2 activation is associated with negative emotions^70^, it is not surprising that just these cerebellar areas exhibit enhanced volume in individuals with anxious attachment.

In addition to increased volumes in lobule VI and Crus 2, Preoccupation with Relationships scores were associated with increased volumes of right mOFC, a prefrontal area critically involved in operational control of emotional and social stimuli^71,72^. More specifically, OFC role in emotion is to decode the reward/punishment goals for action, by representing reward value and transmitting the resulting representations to other brain regions which implement the learning of actions to obtain the reward outcomes signaled by the OFC. Patients with OFC lesions are less sensitive to reward, and are unable to ‘‘think through’’ the consequences of their actions, relying conversely on ingrained habits or immediate information to guide their actions^73,74^. Cerebellar properties of timing, prediction, and learning well integrate with OFC processing to control social and emotional functions^75–77^.

Neuroimaging findings indicate that cerebellum and OFC are both involved in the pathophysiology of psychiatric disorders associated with dysregulation of affect, such as schizophrenia, mood disorders (major depression and bipolar disorder), anxiety disorders (such as phobias), and obsessive–compulsive disorder, post-traumatic stress disorder and attention deficit hyperactivity disorder^71,78,79^. Moreover, while secure attachment is the foundation for psychological well-being^5^, insecure patterns leading to self-doubts, anxiety and distress may represent a risk factor for psychopathology with the specific symptomatology depending on genetic, developmental, and environmental factors^60,80^. Consistently, in adults and adolescents, preoccupied and fearful attachment styles are associated with heightened chronic pain, depression, pain catastrophizing and anxiety^81–83^. The significant relationship between anxious attachment and borderline personality disorder features has been reported in both nonclinical and clinical samples^84^.

In conclusion, we propose that in addition to OFC even the specific cerebellar areas previously demonstrated to be involved in emotional regulation have to be included in the current neurobiological models of human attachment^85^. The present research may represent a step forward in mapping out the attachment process and improving our understanding of the pathophysiology of the attachment-related disorders.

The main strength of the present study is that it is the first macro- and micro-structural (VBM and DTI) study specifically aimed at analyzing the engagement of cerebellar structures in the attachment behavioral system.

Another strength is represented by the rather large sample of non-clinical subjects of both sexes (although exclusively whites) with a wide range of educational level.

However, the current study has some limitations leaving opportunities for future research.

The main limitation of the present research is the application of only a self-report measure of attachment, which may be subject to respondent bias and may potentially over-emphasize attachment as a conscious and detectable process^80^. Although some research suggests self-report measures are reliable and valid sources of participant information^86^, self-reported engagement in attachment-related processes may be of questionable accuracy. Conversely, the usage of informant measures of attachment, such as the Adult Attachment Interview (AAI)^87^, would have allowed evaluating conscious and unconscious memories related to childhood relationships with caregivers as well as assessing the perceived effects of these occurrences on adult personality.

An additional limitation could be that the usage of a VBM correlational approach does not permit to infer causal relationships between brain structural variations and psychological measures, as the ASQ. Furthermore, VBM findings do not allow clarifying the relationships among brain areas potentially involved in the same functions. Finally, the image transformations required for VBM might introduce artifactual volumetric differences, such as a partial volume error. In spite of these limitations, VBM represents a useful approach since it is a user-independent, unbiased exploration of the whole brain.

On such a basis, future studies may benefit from using multi-method approaches to explore the processes underlying the relationship between temperament, attachment and affective stories by using informant measures, interview techniques, and functional neuroimaging methods to capture these complex processes.

## Methods

### Ethical statement

The investigation was carried out in accordance with the latest version of the Declaration of Helsinki. The study design was reviewed by the local ethic committee of the Santa Lucia Foundation IRCCS and the informed consent of all participants was obtained after the nature of the procedures had been fully explained.

### Participants

A sample of 79 healthy subjects (36 males; mean age ± SD: 40.06 ± 12.57 years; range: 19–59; Males: 38.13 y ± 12.24; Females: 41.67 y ± 12.76) belonging to a larger group of healthy volunteers (N = 125), submitted to MRI scan protocol for other studies, were enrolled in the present research. Only those who accepted to come again to Santa Lucia Foundation to be tested on ASQ and the other psychological scales were included in this study. Educational level ranged from an eighth grade to a post-graduate degree (mean education years ± SD: 15.83 ± 2.86; range: 8–25). All participants were right-handed as assessed with the Edinburgh Handedness Inventory^88^. Inclusion and exclusion criteria are described in details in Supplementary Materials section.

### Psychological instruments

#### Attachment style assessment

The Italian version of the Attachment Style Questionnaire (ASQ), a widely used, well-validated, psychometric instrument for a dimensional definition of adult attachment style in normative and clinical populations, was used^89^. The ASQ is based on a self-report questionnaire comprising 40 items answered on a 6-point Likert scale ranging from 1 ("Does not describe me well") to 6 ("Describes me very well"). ASQ has 5 subscales, the first one reflects the secure attachment style, and the remaining 4 ones investigate particular aspects of the insecure attachment style. In more detail, the five subscales are: *Confidence* (8 items) which is associated to secure attachment (Sample item: *I find it relatively easy to get close to other people*); *Discomfort with Closeness* (10 items) *(*Sample item: *I worry about people getting too close*) and *Relationship as Secondary* (7 items) (Sample item: *I find it hard to trust other people*) which are associated to insecure/avoidant attachment; *Need for Approval* (7 items) (Sample item: *It is important to me that others like me)* and *Preoccupation with Relationships* (8 items) (Sample item: *I wonder how I would cope without someone to love me*) which are associated to insecure/anxious attachment. The attachment styles were characterized according to the theoretical models developed by Hazan and Shaver^46^ and by Bartholomew and Horowitz^90^. Internal consistency, test–retest reliability, and factor validity were previously published^89^. In the present study, Cronbach’s α values for the ASQ subscales ranges from 0.66 to 0.79^91^.

#### Depression and anxiety assessment

Presence and severity of depressive symptoms were evaluated by using HAM-D. Scores < 8 indicated no depression, scores from 8 to 17 corresponded to mild depression, scores from 18 to 24 corresponded to moderate depression, and scores > 24 severe depression^49^. Presence and severity of anxiety symptoms were evaluated by using HAM-A, which consists of 14 questions. Scores < 5 indicated no anxiety, scores between 6 and 14 indicated mild anxiety, and score > 14 indicated moderate to severe anxiety^50^. All questionnaires were administered prior to scanning (in general at least one day before).

### Image acquisition

All participants underwent the imaging protocol originally described elsewhere^91–94^. The protocol included standard clinical sequences (FLAIR, DP-T2-weighted), a volumetric whole-brain 3D high-resolution T1-weighted sequence, and a DTI scan protocol, performed with a 3-T Achieva MR imager (Siemens, Erlangen, Germany).

Volumetric whole-brain T1-weighted images were obtained in the sagittal plane using a modified driven equilibrium Fourier transform (MDEFT) sequence (Echo Time/Repetition Time—TE/TR = 2.4/7.92 ms, flip angle 15°, voxel size 1 × 1 × 1 mm^3^). Diffusion volumes were acquired by using echo-planar imaging (TE/TR = 89/8500 ms, bandwidth = 2126 Hz/vx; matrix size 128 × 128; 80 axial slices, voxel size 1.8 × 1.8 × 1.8 mm^3^) with 30 isotropically distributed orientations for the diffusion-sensitizing gradients at one b value of 1000 s mm^2^ and two b = 0 images. Scanning was repeated three times to increase the signal-to-noise ratio. All planar sequence acquisitions were obtained in the plane of the anterior–posterior commissure line. Since the posterior cranial fossa usually falls at the lower limit of the field of view, particular care was taken to center subjects’ head in the head coil, in order to avoid possible magnetic field dishomogeneities or artifacts at the level of the cerebellum.

### Image processing

T1-weighted and DTI images were submitted to several processing steps, described in previous works^91–94^. In brief, T1-weighted images were segmented in order to extract grey matter (GM) maps. Such maps were subsequently normalized, modulated and finally smoothed, before being used for statistical analyses. DTI data were corrected for motion and eddy currents^95^ and normalized before generating Fractional Anisotropy (FA) and Mean Diffusivity (MD) maps. Among DTI indices, MD and FA were used as probes for GM and white matter (WM) micro-structural integrity, respectively^40,91–94^.

In detail, to explore the relationship between regional volumes and empathy on a voxel-by-voxel basis, T1-weighted images were processed and examined using the SPM8 software (Wellcome Department of Imaging Neuroscience Group, London, UK; http://www.fil.ion.ucl.ac.uk/spm), specifically the VBM8 toolbox (http://dbm.neuro.uni-jena.de/vbm.html) running in Matlab 2007b (MathWorks, Natick, MA, USA). The toolbox extends the unified segmentation model^96^ consisting of MRI field intensity inhomogeneity correction, spatial normalization, and tissue segmentation at several pre-processing steps to further improve data quality. Initially, to increase the signal-to-noise ratio, an optimized block-wise nonlocal-means filter was applied to the MRI scans using the Rician noise adaption^97^. Then, an adaptive maximum a posteriori segmentation approach extended by partial volume estimation was employed to separate the MRI scans into GM, WM, and cerebro-spinal fluid. The segmentation step was finished by applying a spatial constraint to the segmented tissue probability maps based on a hidden Markow Random Field model to remove isolated voxels, which unlikely were members of a certain tissue class, and to close holes in clusters of connected voxels of a certain class, resulting in a higher signal-to-noise ratio of the final tissue probability maps. Then, the iterative high- dimensional normalization approach provided by the Diffeomorphic Anatomical Registration through Exponentiated Lie Algebra (DARTEL)^98^ toolbox was applied to the segmented tissue maps to register them to the stereotaxic space of the Montreal Neurological Institute (MNI). The tissue deformations were used to modulate participants’ GM and WM maps to be entered in the analyses. Voxel values of the resulting normalized and modulated GM and WM segments indicated the probability (between 0 and 1) that a specific voxel belonged to the relative tissue. Finally, the modulated and normalized GM and WM segments were written with an isotropic voxel resolution of 1.5 mm^3^ and smoothed with a 6-mm Full-Width Half Maximum (FWHM) Gaussian kernel.

### DTI model

DTI data were pre-processed and analyzed in Explore DTI v4.8.6^94^. Data were corrected for motion and eddy currents. Motion artifacts and eddy current distortions were corrected with B-matrix rotation using the approach of Leemans and Jones^94^. During this processing procedure, all brain scans were rigidly normalized to MNI space during the motion-distortion correction step. A diffusion tensor model was fit at each voxel and maps of FA and MD were generated. All diffusional indexes were finally written in a resolution of 2 × 2 × 2 mm. MD and FA maps were subsequently smoothed by using a Gaussian kernel with a 6-mm FWHM.

MD measures the averaged diffusion of water molecules through tissues providing information on restrictions (e.g., high density of cells) that water molecules encounter. If these obstacles have coherent alignment, on average the water tends to diffuse more along a certain axis. MD reflects cellular and cyto-architectonic changes, which result in higher density of synapses, spines, and capillaries, modifications in the properties of myelin and membranes, alterations in shape of glial cells and neurons. Ultimately, decreased MD reflects increased functional adaptation, and increased MD has been linked to poor cognitive performance or psychiatric symptoms^99^ and to states characterized by reduced efficacy of synaptic and extra-synaptic transmission^100^. FA measures the anisotropy of water diffusion processes and it is positively linked to fiber density, axonal diameter and myelination in WM^101^. Low FA values stand for isotropic diffusion (i.e., unrestricted in all directions), while high FA values indicate diffusion fully restricted along one axis*.*

### Statistical analyses

#### Sociodemographic and psychological variables

Parametric associations between ASQ scores and age, years of formal education, and HAM-D and HAM-A scores, were analyzed by Pearson’s product moment correlations (Fisher’s r to z). Gender differences in ASQ were assessed by unpaired t test. Results of the demographic characteristics were considered significant at the *p* < 0.05 level.

### Volumetric analyses

#### ROI-based VBM

As main aim of the present study we focused the ROI-based VBM on the cerebellum. In the VBM analyses the whole cerebellum has been used as a binary inclusive mask (ROI). Then, on the basis of the cerebellar results and to constrain anatomical hypotheses, we selected several cortical ROIs emerging from previous functional and structural neuroimaging studies. We bilaterally analyzed the orbito-frontal cortex (BA11, BA47)^21,26,28,51^, middle frontal area (BA 9, BA10)^27,28^, insula^28,36^, and cingulate cortex ^21^.

The MNI-oriented atlas of the human brain (Automated Anatomical Labeling Atlas, AAL)^102^ was used to extract GM masks of the ROIs singularly achieved by meaning all GM probability maps, obtained in the VBM8 processing steps, thresholding the relative image to a value of 0.3 (i.e. removing all voxels having a probability to belong to GM lower or equal to 29%), and manually removing all the other structures (e.g. for the cerebellum by manually removing all the non-cerebellar structures) using the AAL template, as reference. The resulting data were then fed into VBM analyses to evaluate morphological changes associated with ROIs and attachment subscales. We evaluated at the voxel-level the associations between cerebellar or neocortical structural measures and ASQ scores, by using SPM8 within the framework of the General Linear Model. Multiple-regression analyses were computed by singularly using the measures of ROIs GM volumes as dependent variables, the scores of ASQ subscales as regressors. Moreover, when significantly associated to attachment ASQ subscales, also age, gender, education years, depression or anxiety levels were used as covariates. Gender was always considered a ‘‘dummy variable’’ given its dichotomic nature. We considered significant only the relationships whose voxels were part of a spatially contiguous cluster size of a minimum of 50 voxels, and that survived (*p* < 0.05) at the Family Wise Error (FWE) correction.

To obtain the precise anatomical localization of VBM results, we superimposed statistical maps onto Diedrichsen’s probabilistic atlas of the human cerebellum, which subdivides the cerebellum into ten different regions^103^. For extra-cerebellar cortical ROIs the AAL template was used. Since the existing maps of the OFC differ with respect to number of areas identified, relative size, extent and spatial relationship to each other^104^, we referred to both MNI coordinates and BAs to avoid confusing classifications.

#### DTI analyses

The areas significantly associated (p_FWEcorr_) with ASQ subscales at macro-structural analyses (cerebellar lobules VI and Crus 2 and mOFC) were used as masks and applied to MD and FA maps, to extract mean micro-structural values for each measure. Parametric associations between attachment scores and mean MD or FA values were analyzed by Pearson’s product moment correlations (Fisher’s r to z) to assess potential significant associations also with micro-structural measures. Analyses were also controlled for significantly associated socio-demographic variables.

## Supplementary Information

Supplementary Information.
