# Supplementary material for: Cerebellar engagement in the attachment behavioral system
Source: Sci Rep. 2022 Aug 9;12:13571. doi: 10.1038/s41598-022-17722-x (PMC9363408; doi:10.1038/s41598-022-17722-x)
Supplement: Supplementary file 1 — Supplementary Information. [file 41598_2022_17722_MOESM1_ESM.docx]

**Supplementary Materials**

**Participants’ inclusion and exclusion criteria**

Inclusion criteria were: age between 18 and 65 years and suitability for MRI scanning. Exclusion criteria included: (1) cognitive impairment or dementia, based on Mini Mental State Examination (MMSE) (Folstein et al., 1975) scores ≤ 24 (Measso et al., 1993), and confirmed by clinical neuropsychological evaluation by using the Mental Deterioration Battery (Carlesimo et al., 1996) and the NINCDS-ADRDA criteria for dementia (McKhann et al., 2011); (2) subjective complaint of memory difficulties or of any other cognitive deficit, regardless of interference with daily activities; (3) major medical illnesses, e.g. diabetes (not stabilized), obstructive pulmonary disease, or asthma; hematologic and oncologic disorders; pernicious anemia; clinically significant gastrointestinal, renal, hepatic, endocrine, or cardio-vascular system diseases; newly treated hypothyroidism; (4) current or reported mental (assessed by SCID-I and the SCID-II) (First & Pincus, 2002) or neurological (assessed by clinical neurological evaluation) disorders (e.g. schizophrenia, mood disorders, anxiety disorders, stroke, Parkinson’s disease, seizure disorder, head injury with loss of consciousness, and any other significant mental or neurological disorder); (5) known or suspected history of alcoholism or drug dependence and abuse, evaluated by structured interviews (SCID I or SCID II) (First & Pincus, 2002; Spitzer, 1992); (6) MRI evidence of focal parenchymal abnormalities or cerebro-vascular diseases: for each subject, a trained neuroradiologist and a neuropsychologist expert in neuroimaging co-inspected all the available clinical MRI sequences (i.e. T1- and T2-weighted and FLAIR images) to ensure that the subjects were free from structural brain pathologies and vascular lesions (i.e. FLAIR or T2-weighted hyper-intensities and T1-weighted hypo-intensities).

**References**

Carlesimo, G.A., Caltagirone, C., Gainotti, G., Fadda, L., Gallassi, R., Lorusso, S., Marfia, G., Marra, C., Nocentini, U., Parnetti, L., 1996. The Mental Deterioration Battery: Normative Data, Diagnostic Reliability and Qualitative Analyses of Cognitive Impairment. Eur Neurol 36, 378–384. https://doi.org/10.1159/000117297

First, M.B., Pincus, H.A., 2002. The *DSM-IV Text Revision:* Rationale and Potential Impact on Clinical Practice. PS 53, 288–292. https://doi.org/10.1176/appi.ps.53.3.288

Folstein, M. F., Folstein, S. E. & McHugh, P. R. “Mini-mental state”. Journal of Psychiatric Research 12, 189–198 (1975).

McKhann, G.M., Knopman, D.S., Chertkow, H., Hyman, B.T., Jack, C.R., Kawas, C.H., Klunk, W.E., Koroshetz, W.J., Manly, J.J., Mayeux, R., Mohs, R.C., Morris, J.C., Rossor, M.N., Scheltens, P., Carrillo, M.C., Thies, B., Weintraub, S., Phelps, C.H., 2011. The diagnosis of dementia due to Alzheimer’s disease: Recommendations from the National Institute on Aging-Alzheimer’s Association workgroups on diagnostic guidelines for Alzheimer’s disease. Alzheimer’s & Dementia 7, 263–269. https://doi.org/10.1016/j.jalz.2011.03.005

Measso, G., Zappalà, G., Cavarzeran, F., Crook, T.H., Romani, L., Pirozzolo, F.J., Grigoletto, F., Amaducci, L.A., Massari, D., Lebowitz, B.D., 1993. Raven’s colored progressive matrices: a normative study of a random sample of healthy adults. Acta Neurol. Scand. 88, 70–74. https://doi.org/10.1111/j.1600-0404.1993.tb04190.x

Spitzer, R.L., 1992. The Structured Clinical Interview for DSM-III-R (SCID): I: History, Rationale, and Description. Arch Gen Psychiatry 49, 624. https://doi.org/10.1001/archpsyc.1992.01820080032005

**Attachment Style Questionnaire**

_____________________________________________________________________________

Show how much you agree with each of the following items by rating them on this scale:

1 = totally disagree; 2 = strongly disagree; 3 = slightly disagree; 4 = slightly agree; 5 = strongly agree; 6 = totally agree.

_____________________________________________________________________________

Confidence 1. Overall, I am a worthwhile person (*Nel complesso sono una persona valida*).

Confidence 2. I am easier to get to know than most people (*E’più facile arrivare a conoscere me che la maggior parte delle altre persone*).

Confidence 3. I feel confident that people will be there for me when I need them (*Sono fiducioso che gli altri ci saranno quando avrò bisogno di loro*).

Discomfort 4. I prefer to depend on myself rather than other people (*Preferisco dipendere da me stesso che dagli altri*).

Discomfort 5. I prefer to keep to myself (*Preferisco stare sulle mie*).

R as S 6. To ask for help is to admit that you're a failure (*Chiedere aiuto vuol dire ammettere di essere un fallimento*).

R as S 7. People's worth should be judged by what they achieve (*Il valore di una persona andrebbe giudicato in base ai suoi successi).*

R as S 8. Achieving things is more important than building relationships (*Raggiungere gli obbiettivi è più importante che andare d’accordo con gli altri*).

R as S 9. Doing your best is more important than getting on with others (*Dare il massimo è più importante che andare d’accordo con gli altri*).

R as S 10. If you've got a job to do, you should do it no matter who gets hurt (*Se hai un lavoro da fare, non dovrebbe importarti chi ne avrà un danno*).

N for A 11. It's important to me that others like me (*Per me è importante piacere agli altri*).

N for A 12. It's important to me to avoid doing things that others won't like (*Per me è importante evitare di fare cose che agli altri non piacciono*).

N for A 13. I find it hard to make a decision unless I know what other people think (*Trovo difficile prendere una decisione, a meno che non sappia ciò che pensano gli altri*).

R as S 14. My relationships with others are generally superficial (*Le mie relazioni con gli altri sono solitamente superficiali*).

N for A 15. Sometimes I think I am no good at all (*A volte penso di non valere nulla*).

Discomfort 16. I find it hard to trust other people (*Ho difficoltà a fidarmi degli altri*).

Discomfort 17. I find it difficult to depend on others (Ho difficoltà a dipendere dagli altri).

Preoccupation 18. I find that others are reluctant to get as close as I would like (*Trovo che gli altri siano riluttanti a entrare in confidenza quanto io vorrei*).

Confidence 19. I find it relatively easy to get close to other people (*Trovo relativamente facile entrare in confidenza con gli altri*)

Discomfort (R) 20. I find it easy to trust others (*Mi fido facilmente degli altri*).

Discomfort (R) 21. I feel comfortable depending on other people (*Mi trovo a mio agio nel dipendere dagli altri*).

Preoccupation 22. I worry that others won’t care about me as much as I care about them (*Mi preoccupo che agli altri non importerà di me quanto a me importa di loro*)

Discomfort 23. I worry about people getting too close (*Mi preoccupo quando la gente entra troppo in confidenza con me*).

N for A 24. I worry that I won't measure up to other people (*Mi preoccupo di non essere all’altezza degli altri*).

Discomfort 25. I have mixed feelings about being close to others (*Ho sentimenti contrastanti circa l’essere in confidenza con gli altri*).

Discomfort 26. While I want to get close to others, I feel uneasy about it (*Se da un lato voglio entrare in confidenza con gli atri, dall’altro mi sento a disagio*).

N for A 27. I wonder why people would want to be involved with me (*Mi chiedo perchè la gente voglia avere a che fare con me*).

Preoccupation 28. It's very important to me to have a close relationship (*Per me è veramente importante avere una relazione stretta*).

Preoccupation 29. I worry a lot about my relationships (*Mi preoccupo molto delle mie relazioni*).

Preoccupation 30. I wonder how I would cope without someone to love me (*Mi chiedo come me la caverei senza qualcuno che mi ama)*.

Confidence 31. I feel confident about relating to others (*Mi sento fiducioso nelle relazioni con gli altri*).

Preoccupation 32. I often feel left out or alone (*Spesso mi sento lasciato in disparte o da solo*).

Confidence (R) 33. I often worry that I do not really fit in with other people (*Spesso mi preoccupo di non riuscire ad entrare in sintonia con gli altri)*.

Discomfort 34. Other people have their own problems so I don’t bother them with mine (*Gli altri hanno i loro problem per cui non li infastidisco con i miei*).

N for A 35. When I talk over my problems with others, I generally feel ashamed or foolish (*Quando discuto dei miei problemi con gli altri, di solito mi vergogno o mi sento stupido*).

R as S 36. I am too busy with other activities to put much time into relationships (*Sono troppo impegnato in altre attività per dedicare molto tempo alle relazioni*).

Confidence 37. If something is bothering me, others are generally aware and concerned (*Se qualcosa mi disturba, gli altri solitamente ne sono consapevoli e preoccupati*).

Confidence 38. I am confident that other people will like and respect men (*Sono fiducioso di essere gradito e rispettato dagli altri*).

Preoccupation 39. I get frustrated when others are not available when I need them (*Mi sento frustrato quando gli altri non sono disponibili nel momento in cui ne ho bisogno*).

Preoccupation 40. Other people often disappoint me (*Gli altri spesso deludono le mie aspettative*).

_______________________________________________________________________________Abbreviations: R as S = Relationships as Secondary; N for A = Need for Approval;

Items marked (R) need to be reverse-scored; In brackets, the Italian translation in italics.
